# Membrane Separation Processes in Wastewater and Water Purification, Volume II

**DOI:** 10.3390/membranes14060119

**Published:** 2024-05-22

**Authors:** Alexandre Giacobbo, Andréa Moura Bernardes

**Affiliations:** Post-Graduation Program in Mining, Metallurgical and Materials Engineering (PPGE3M), Federal University of Rio Grande do Sul (UFRGS), Av. Bento Gonçalves n. 9500, Agronomia, Porto Alegre 91509-900, RS, Brazil

## 1. Introduction

Water is a crucial natural resource, essential for the development of a range of human activities, from agricultural and industrial to domestic; therefore, its availability is associated with a region or country’s economic growth [1,2]. Nevertheless, the indiscriminate use of this good can cause its scarcity, whether in terms of quality, quantity, or both, putting at risk the living conditions of future generations [3,4].

Currently, 2.2 billion people do not have access to drinking water, around 1.5 billion face shortages year-round, and 4 billion—that is, half of the world’s population—live under severe water scarcity during at least one month each year [5]. This scenario is even more worrying given that the demand for water is constantly increasing due to rapid population growth, urbanization, consumerist culture, and rising pressure from agriculture, industry, and the energy sector. According to information from the UNDP, 700 million people could be displaced from their homelands by 2030 due to water scarcity [6].

These factors are aggravated by the fact that a large part of wastewater is discarded into the environment without undergoing adequate treatment or even without any treatment at all, contaminating the available water resources [6,7]. Nowadays, the disposal of untreated wastewater is unacceptable, as, in addition to polluting natural water resources, which makes their use *in natura* unfeasible and the treatment of this water more expensive, it also represents a waste of resources, going against the current economic gold standard, which advocates for circular economy practices [8]. Furthermore, it is already widely known that in circumstances of water scarcity, wastewater is considered a valuable water resource; as previously stated, the global situation of water scarcity has deteriorated over the years. Also, within the scope of the circular economy and sustainable development, wastewater can be considered a crucial renewable resource [9]. In this regard, data in the literature point to an increase in wastewater reuse in recent decades in various parts of the world [2,10,11]. In California, for example, wastewater reuse doubled from 1970 to 2001 [12]. In 2010, 20% of wastewater was reused and the remainder was discarded into the ocean; a reversal of this scenario, in which 80% of wastewater will be reused and only 20% disposed of, is expected by 2030 [13].

In fact, several factors, such as the water crisis, tax policies, stricter regulations, and environmental awareness, have boosted the reuse of wastewater. Moreover, various technological and innovation aspects have been identified as drivers of water reuse practices; these include the development of new technologies and treatment processes, the optimization of processes and operational conditions, the integration of different technologies, and innovations implemented in existing technologies and processes, as well as the study and development of new materials.

In this context, since the early 1960s, with the development of the Loeb–Sourirajan process to produce anisotropic reverse osmosis membranes for desalination, research into the field of membranes has increased immensely [14]. Since then, membrane separation processes have been gaining more and more prominence, not only as a desalination technology but also in water treatment for potabilization, hardness softening, production of ultrapure water, and the treatment of a wide range of wastewater types, as well as in separation processes in the chemical, pharmaceutical, and biotechnology industries, among others.

Indeed, the success of a wide range of applications and the increasing expansion of the usage of membrane separation processes, when compared to conventional (waste)water treatment or separation processes, are associated not only with decreased membrane production costs but also with the inherent characteristics of these technologies, which include low energy consumption, fewer process steps, ease of scale-up, small footprint, high separation efficiency, and high quality of the final product, in addition to ensuring greater safety of the process as a whole.

With these advantages in mind, in 2020, we launched the first Special Issue of *Membranes*, entitled “Membrane Separation Process in Wastewater and Water Purification” [15], in which we intended to cover recent developments and advances in all aspects related to membrane-based processes for obtaining pure and ultrapure water, treatment of brines and hypersaline solutions, development of new materials for water or wastewater purification, development/integration of processes for wastewater and water purification, concentration polarization and fouling, and recovery of water or resources. Six research papers were published in the first Special Issue:

- The Effect of pH on Atenolol/Nanofiltration Membranes Affinity, doi:10.3390/membranes11090689;
- Application of Coagulation–Membrane Rotation to Improve Ultrafiltration Performance in Drinking Water Treatment, doi:10.3390/membranes11080643;
- Fabrication of Cementitious Microfiltration Membrane and Its Catalytic Ozonation for the Removal of Small Molecule Organic Pollutants, doi:10.3390/membranes11070532;
- Study of the Ecological Footprint and Carbon Footprint in a Reverse Osmosis Sea Water Desalination Plant, doi:10.3390/membranes11060377;
- Direct Purification of Digestate Using Ultrafiltration Membranes: Influence of Pore Size on Filtration Behavior and Fouling Characteristics, doi:10.3390/membranes11030179;
- Oily Water Separation Process Using Hydrocyclone of Porous Membrane Wall: A Numerical Investigation, doi:10.3390/membranes11020079.

In 2022, likewise looking to collate publications that encompass recent developments and advances in all aspects related to membrane-based processes, we launched Volume II. In total, five articles were published in this Special Issue, and they are briefly described in the coming paragraphs. This Editorial does not intend to elaborate on each of the articles but rather to encourage you, the reader, to delve into them.

## 2. Articles Published in this Special Issue

Nasution and collaborators (Contribution 1) investigated the development of PVDF and tin dioxide hybrid membranes. The manufacturing process was assisted by electric field treatment in order to homogenize the distribution of tin dioxide particles in the polymeric matrix, in addition to reducing porosity and increasing the resistance and resilience of the membranes.

Contribution 2 (Qasim et al.) deals with the simulation and fabrication of anodic aluminum oxide (AAO) membranes and their application as microfiltration for fluid purification. The authors assessed a system with two layers of AAO membranes with different pore sizes, achieving an effective purification of fluids. In fact, the authors report that the main applications of the developed membranes and of the evaluated system are in microfluidic devices for fluid filtration, blood filtration, and biomedical analysis. Nevertheless, they also point to these systems as potential candidates for the filtration of water and other liquids.

Alebrahim and Moreau (Contribution 3) fabricated photocatalytic microfiltration and ultrafiltration membranes decorated with titanium dioxide using the suspension plasma spray process. The thickness of the membranes was controlled by the number of plasma spray passes (up to 12 cycles) over the surface of the substrates. They evaluated the membranes for filtration performance using silica particles of different sizes and polyethylene oxide with molecular weights from 20 kDa to 1000 kDa as well as fouling parameters and self-cleaning capacity under visible light by filtering solutions with organic pollutants, such as humic acid and methylene blue dye. They demonstrated that the rejection rate rises as membrane thickness increases, especially for ultrafiltration membranes, in which larger-surface pores are filled with agglomerates of titanium dioxide nanoparticles. Furthermore, the photocatalytic activity of titanium dioxide nanoparticles was effective in recovering the flux for both membranes under visible light illumination.

The leaching of pollutants from contaminated soil and sludge and the resulting water contamination are also a matter of concern. From this perspective, Contribution 4, by Hadi and Awadh, deals with the electro-kinetic remediation of chromium-polluted sludge with and without the use of membranes. The apparatus used in their study consists of an electrochemical cell with three compartments (anodic, central, and cathodic), filled, respectively, with deionized water at pH ≈ 10, chromium-contaminated sludge, and acetic acid at pH ≈ 3. Graphite electrodes coupled to a power source were used, and anion and cation exchange membranes were placed close to the anode and cathode compartments, respectively. Under the best operating conditions, the study accomplished chromium removal rates greater than 80%, illustrating that the electro-kinetic remediation of toxic-metal-contaminated sludge with ion-selective membranes is a promising technique and paving the way for the development of further studies to enable its large-scale application.

Sustainable development has become a guiding principle for companies and organizations; not only does it allow them to continuously improve, but may also make or break their position on the market. In light of this, the article by Alvizuri-Tintaya and co-workers (Contribution 5) deals with the mathematical and statistical evaluation of reverse osmosis in the removal of manganese as a way to achieve sustainable operating parameters. In their study, the authors raise the problems associated with manganese mining, such as water contamination, health risks, and forms of water treatment to remove this metal, focusing on reverse osmosis treatment. They demonstrate that it is possible to remove manganese with reverse osmosis at low pressures, thus reducing the operating costs of the treatment system. This opens up the possibility of implementing reverse osmosis in contexts where water contamination problems and economic limitations are present.

## 3. Conclusions and Future Directions

This compilation of articles covers research devoted not only to using membrane separation processes for waste(water) purification but also to the development of membranes with special properties or for specific applications, as in the cases of Contribution 3, which deals with TiO_2_ photocatalytic membranes with self-cleaning capacity, Contribution 2, which investigates a microfiltration device using a dual anodic aluminum oxide membrane, and Contribution 1, which assesses the application of an electric field in the manufacture of PVDF–tin dioxide membranes. Contribution 5, in turn, addresses a current and important topic in which membrane-based processes have shown prominence, namely sustainability in water treatment systems. Finally, an impressive article (Contribution 4) explores the electro-kinetic technique with ion-selective membranes for removing toxic metals from contaminated soils and sludge.

There are still many aspects to be improved in the field of membranes and membrane separation processes. Based on the articles published in this Special Issue, we identified the following directions:

- Studies report that tin dioxide has photocatalytic [16] and biocidal [17] activity; therefore, it is important to further investigate the photo-self-cleaning and antifouling capabilities of polymeric membranes decorated with this material. Different quantities and sizes of tin dioxide (nano)particles in different polymer matrices can also be evaluated. The same principle could be used for other types of (nano)particles, such as TiO_2_, Ag, and Cu, among others. Furthermore, the treatment of membranes with electric fields also deserves further studies;
- Although they have been indicated for possible water filtration applications, devices with dual anodic aluminum oxide membranes have found widespread use in microfluidic separation and purification, suggesting that its potential use for a wide range of real fluids should also be tested. Furthermore, experiments should be conducted on different combinations of membranes and operating conditions;
- From the perspective of the 2030 Agenda, discussing the sustainability of membrane separation processes in (waste)water treatment is imperative. Although one of the articles in this Special Issue addresses this matter, there is still much to be explored. Future studies on this matter will contribute to a greater application of membrane technologies;
- The usage of membranes in the electro-kinetic remediation of contaminated soils and sludge opens up a vast field of research. For instance, the evaluation of other types of membranes (homogeneous and heterogeneous) that can be manufactured from several materials, the assessment of other anodic and cathodic solutions, as well as variations in operating conditions, electrochemical cell geometry, and target contaminants merit consideration in the future.
